# Investigation on the water-conducting zone evolution in weak overburden due to repeated mining of thick coal seams

**DOI:** 10.1038/s41598-026-46365-5

**Published:** 2026-07-02

**Authors:** Zhenqiang Yang, Xingguo Niu, Yuhao Feng, Daohan Li, Hongfan Xu, Siwei Jin

**Affiliations:** 1 College of Energy and Transportation Engineering, Inner Mongolia Agricultural University, Inner Mongolia Hohhot, 010018 China; 2 Inner Mongolia Nonferrous Geological Mining (Group) Comprehensive Survey Co., Ltd, Inner Mongolia Hohhot, 010010 China; 3 School of Mechanics and Civil Engineering, China University of Mining & Technology (Beijing), Beijing, 100083 China

**Keywords:** Thick coal seam, Repeated mining, Fracture zone, Weak overburden, Key stratum theory, Energy science and technology, Engineering, Solid Earth sciences

## Abstract

Based on the II_2-1_ and II_3_ coal seams in China’s Lingdong Coal Mine, the activation mechanism of water-conducting fractures in weak overburden under repeated mining of thick coal seams are studied by theoretical analysis, numerical simulation and field measurement in this paper. The main findings include that the key stratum theory and the critical fracturing span of the overlying rock layers were combined to determine the theoretical height of the water-conducting fracture zone in the II_3_ coal seam under repeated mining disturbance as 83 m. UDEC numerical simulations revealed that as the working face advanced, fractures in the overburden above the II_2-1_ and II_3_ coal seams initially propagated upward following roof fracturing; after advancing a specific distance, fractures in the collapsed strata at the center of the goaf began closing and compacting; with continued mining operations, the working face gradually attained sufficient mining conditions while these mining-induced fractures entered a stable compaction stage, ultimately resulting in the water-conducting fracture zone exhibiting a characteristic distribution pattern where fractures prominently developed above the open-off cut and stopping line yet remained closed within the central goaf area, with its height stabilizing at approximately 78 m. Field measurements indicated that the actual height of the water-conducting fractures in the II_3_ coal seam overburden ranged from 74.5 m to 77.1 m, averaging 75.8 m. Comprehensive results demonstrate that the height of water-conducting fracture zones in weak overburden is significantly lower than the failure height observed in conventional sandstone formations. These findings provide theoretical references for safe coal mining under water bodies.

## Introduction

Coal mining causes failure in the overlying rock strata, leading to the formation of a fracture zone in the overburden. When the fracture zone connects to surface water bodies or underground aquifers, water inrush hazards may happen. There are about 285 key coal mines often suffer from serious water inrush disasters during coal extraction^1,2^. The total coal reserves threatened by water inrush are estimated at 25 billion tons^3^. Mining induced water-conducting zone height prediction has been the topic for several decades. The height of water-conducting zone is closely related to mechanical properties of overlying rock strata. Some prediction methods have been proposed by researchers, but most of them are empirical prediction methods based on field measured data^4–7^. The key stratum theory, proposed by Qian^8^, indicates that there are some stiff and thick rock strata in overburden, which control rock strata movement and influence the height of water-conducting zone^9,14^. In weakly cemented overburden, however, the controlling role of strata may not be governed solely by conventional stiffness-dominant behavior, but also by deformation compatibility, bending resistance and the limitation imposed by the available void space beneath the strata. In addition, the height of water-conducting zone is also influenced by mining methods and mining face length^11^. An enhanced empirical prediction formula has been proposed that is applicable to fully mechanized top-coal caving mining under different overburden strengths^10,12^. Multi-seam mining will cause repeated damage of overburden, and a quantitative relationship between water-conducting zone height and cumulative coal seam thickness is developed^13^. Ultra-thick coal seams exist in some mining areas, large-scale mining will significantly increase the water-conducting zone height of overburden and the risks of roof water inrush^16–18^. And steeply inclined ultra-thick coal seam mining induced water-conducting zone height is also investigated^15^. Furthermore, numerical simulation and on-site measurement are useful methods on revealing the development law of water-conducting zones^19–23^. The concepts of energy conversion, kinetic energy calculation, and a series of computer methods also provide new technical perspectives for predicting the development height of water-conducting fracture zones^24–31^. Accurate prediction of water-conducting zones of overburden strata is critical for mine water hazard prevention and safe mining.

At present, numerous scholars have conducted a series of studies on the development characteristics of water-conducting fractures in overlying strata, which provides crucial theoretical references for this research. However, the evolution laws of water-conducting fracture zones in weak overburden under repeated mining of thick coal seams in the Lingdong Coal Mine remain unresearched. Therefore, based on the hydro-geological conditions of Lingdong Coal Mine, theoretical analysis, numerical simulation and field measurements are adopted to investigate the development mechanisms of water-conducting fractures in overburden under repeated mining impacts, which holds significant theoretical importance and engineering application value for providing theoretical support for safe multi-seam mining under similar aquifers.

## Engineering background

**Fig. 1:**
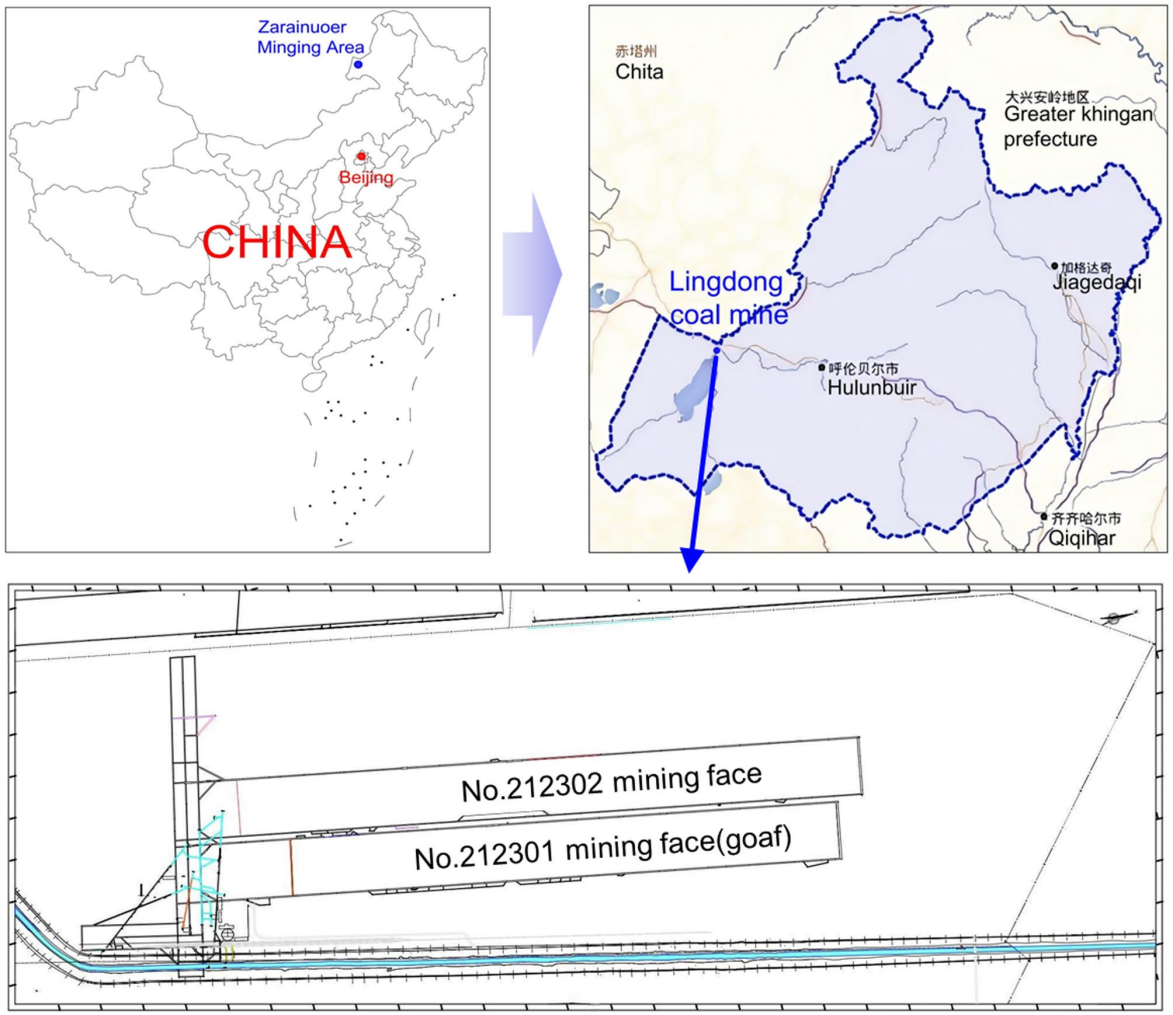
Lingdong coal mine location and studied mining face.

Lingdong coal mine is located in Inner Mongolia of China. The study area contains two coal seams, with an average vertical separation of approximately 120 m. Due to previous mining of the upper II_2-1_ coal seam, a lake has formed on the surface. Additionally, the Xinkai River is located near the mine, as shown in Fig. 1. The below II_3_ coal seam mining is threatened by the ground water and the possible water of upper goafs. When the fracture zone induced by II_3_ coal seam mining connects to surface water bodies, water inrush hazards may happen. The No. 212,301 mining face is the first one of II_3_ coal seam mining and the mining thickness of coal seam is approximately 12 m.

The 212,302 upper working face of the II_3_ coal seam is located in the north wing of the West No.1 mining district. The working face has a strike length of approximately 3020 m and a dip width of 276 m. The elevation of the working face ranges from + 72.9 m to + 131 m, while the surface elevation above the panel varies between + 545 m and + 547 m, resulting in a burial depth of approximately 410–470 m. The average thickness of the II_3_ coal seam is 24.18 m. Sublevel fully mechanized top-coal caving mining was adopted. The total mining thickness of the upper sublevel was approximately 12 m, achieved with a cutting height of 3.8 m and a top-coal caving height of about 8.2 m, giving a cutting-to-caving ratio of approximately 1:2. The upper II_2-1_ coal seam, with an average thickness of approximately 15 m, had been mined previously. The vertical distance between the II_2-1_ and II_3_ coal seams ranges from 90 m to 120 m. During the extraction of the II_2-1_ seam, a 20 m-wide coal pillar was retained locally. The II_3_ working face is partially located beneath the previously mined-out goaf of the II_2-1_ seam and partially beneath the residual coal pillar. Therefore, the overburden above the II_3_ seam experienced repeated mining disturbance, forming a typical repeated mining condition.

**Fig. 2:**
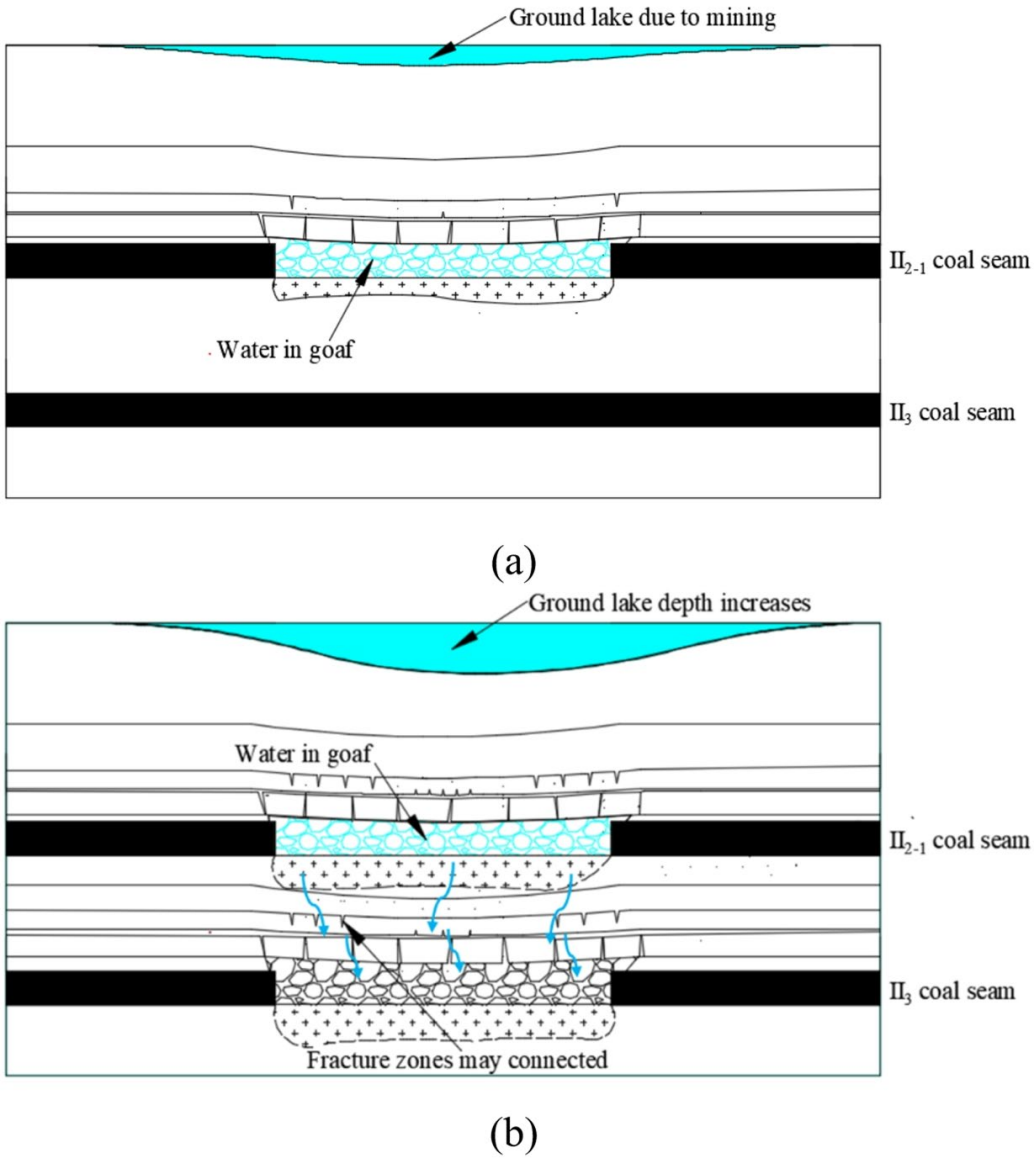
Diagram of repeated coal mining induced water inrush.

According to traditional empirical prediction methods, the water-conducting zone due to II_3_ coal seam mining will develop upward to the goaf of II_2−1_ coal seam, as shown in Fig. 2. However, according to borehole lithological statistics of the bedrock column above the II_3_ coal seam, mudstone-dominated strata account for approximately 70% of the total overburden thickness in the vertical direction. Mineralogical analysis indicates that the clay mineral content in the mudstone reaches about 60.5%, with illite as the dominant component. Such weakly cemented mudstone exhibits pronounced water-induced swelling and softening characteristics, which contribute to the reduction of fracture connectivity. This lithological characteristic plays a key role in controlling the upward development of the water-conducting fracture zone under repeated mining conditions. The mudstone-dominated overburden can effectively inhibit the formation of persistent hydraulic pathways into the goaf. Therefore, accurately determining the height of the water-conducting fracture zone is of great importance.

## Theoretical analysis of water-conducting zone height caused by repeated thick coal seam mining

### Determination of key strata and critical breaking span

There are some key strata in overburden, which can control rock strata movement. Based on the key stratum theory, proposed by Qian, the load acting on the key strata can be calculated by^33^:

1 $${\left. {{{\mathrm{q}}_1}(x)} \right|_m}=\frac{{{E_1}h_{1}^{3}\sum\limits_{{i=1}}^{m} {{\gamma _i}{h_i}} }}{{\sum\limits_{{i=1}}^{m} {{E_i}h_{i}^{3}} }}$$

In the formulation, ${\left. {{q_1}(x)} \right|_m}$denotes the load transferred from the No. *m* stratum to the primary hard-thick stratum, where *h*_*i*_, *γ*_*i*_, *E*_*i*_ represent the thickness, unit weight, and elastic modulus of the No. *i* stratum (i = 1, 2, …, m), respectively. Accounting for the additional load applied by the No. (*m* + 1) stratum on the primary hard-thick stratum, the total load is expressed as:

2 $${\left. {{q_1}(x)} \right|_{m + 1}} = \frac{{{E_1}h_1^3\sum\limits_{i = 1}^{m + 1} {{\gamma _i}{h_i}} }}{{\sum\limits_{i = 1}^{m + 1} {{E_i}h_i^3} }}$$

Since the No. (*m* + 1) stratum is a thick and hard rock layer with its deflection smaller than that of the underlying strata, the rock layers above the No. (*m* + 1) layer no longer require the lower rock layers to bear the loads they bear. Therefore, the following condition must be satisfied:

3 $${\left. {{q_1}(x)} \right|_{m + 1}} < {\left. {{q_1}(x)} \right|_m}$$

Substitution the Eqs. (1) and (2) into Eq. (3), we can obtain the following equation:

4 $${\gamma _{m + 1}}\sum\limits_{i = 1}^m {{E_i}h_i^3} < {E_{m + 1}}h_{m + 1}^2\sum\limits_{i = 1}^m {{h_i}{\gamma _i}}$$

Equation (4) serves as the formula for determining the position of thick-hard stratum. Starting from the first hard thick stratum, the position of the second hard stratum is determined using the aforementioned approach. This process continues until the uppermost hard stratum is identified. It should be noted that the above criterion originates from the conventional key-stratum theory developed mainly for relatively competent overburden. In the present study area, however, the overburden is dominated by weakly cemented mudstone-rich strata. Therefore, the identified “key strata” should be understood as controlling strata within a weak-overburden system, whose controlling effect is not determined solely by high stiffness and strength, but also by their thickness, deformation coordination with adjacent strata, and the restriction imposed by the underlying free space during mining-induced movement.

According to Elastic mechanics, the critical breaking span of the rock strata beam can be derived as follows^34^:

5 $${L_k} = {h_k}\sqrt {\frac{{2{\sigma _t}}}{{{q_k}}}}$$

In the equation, *h*_*k*_ denotes the thickness of the No. *k* hard stratum (m); ${\sigma _k}$represents the tensile strength of the No. *k* hard stratum (MPa); and ${q_k}$is the load sustained by the No. *k* hard stratum. Based on Eq. (1), ${q_k}$ can be determined by the following formula:

6 $${q_k} = \frac{{{E_{k,0}}h_{k,0}^3\sum\limits_{j = 0}^{{m_k}} {{h_{k,j}}{\gamma _{k,j}}} }}{{\sum\limits_{j = 0}^{{m_k}} {{E_{k,j}}h_{k,j}^3} }}$$

In the equation, the subscript *k* denotes the No. *k* hard stratum; the subscript *j* represents the layer index of the soft stratum group governed by the No. *k* hard stratum; and *m*_*k*_ indicates the number of soft strata controlled by the No. *k* hard stratum.

If the criterion ${L_k} < {L_{k + 1}}$ is satisfied, the No. *k* hard stratum is identified as the key stratum, and its critical span must be less than those of all overlying hard strata. Based on the mechanical model of the overlying key stratum after initial fracture, the critical advance distance of the working face at the fracture of each key stratum is determined as follows:

7 $${L_{G,j}} = \sum\limits_{i = 1}^m {{h_i}\cot {\varphi _q}} + {l_{G,j}} + \sum\limits_{i = 1}^m {{h_i}\cot {\varphi _h}}$$

In the equation, *L*_*G, j*_ denotes the face advance distance when the No. *j* key stratum fractures; *m* represents the number of rock strata from the coal seam roof to the No. *j* key stratum; *h*_*i*_ signifies the thickness of the No. *i* rock stratum; *l*_*G, j*_ is the critical span at the initial fracture of the No. *j* key stratum without support from underlying strata; ${\varphi _q}$and ${\varphi _h}$denote the front and rear fracture angles of the rock stratum, respectively.

### Bending deformation of soft rock strata

Based on geological drilling data from Lingdong Coal Mine, approximately 70% of the overburden strata in this mining district comprise mudstone. Core samples obtained from the upper return air channel drill hole of 212,302 further confirmed that the majority of the overlying layers are poorly cemented mudstone, belonging to weakly consolidated, low-strength strata. An analysis of horizontal tensile deformation in the weak overburden after mining indicates that the height at which this deformation reaches a critical value corresponds to the vertex of the “two zones” (the caving zone and the fractured zone). Therefore, determining the vertex position of the water-conducting zone by applying the critical horizontal tensile deformation value of the rock layer should be a feasible method. Under such weak-overburden conditions, the controlling role of a stratum is more appropriately reflected by its bending stability and deformation compatibility than by a purely brittle “hard roof” criterion. Accordingly, some relatively thick mudstone layers may still act as controlling strata by accommodating mining-induced deformation without immediate fracture, thereby limiting the upward propagation of the water-conducting fractured zone. The formula for calculating the free space height beneath each stratum layer is as follows:

8 $${\Delta _i} = M - \sum\limits_{j = 1}^{i - 1} {{h_j}} ({k_j} - 1)$$

In the equation ${\Delta _i}$ represents the free space height beneath the No. *i* rock stratum; *M* denotes the mining thickness of the coal seam; *h*_*j*_ signifies the thickness of the No. *j* rock stratum; *k*_*j*_ is the bulking factor of the No. *j* rock layer.

When the working face advances to the critical distance that induces peak tensile strain, the maximum deflection of the rock stratum is expressed as follows:

9 $${\omega _{\max }} = \frac{{5q{l^4}}}{{384EI}}$$

At this point, *ω*_*i*,max_ > *∆*_*i*_ when the maximum deflection of the soft stratum exceeds the height of its underlying void space, the soft stratum will remain in a plastic state and will not fail due to the limitation of the void space. Consequently, the water-conducting zone will cease to develop upward. Conversely, if the maximum deflection of the soft stratum is less than the height of its underlying void space, a failure will occur, leading to the formation of water conduction.

**Fig. 3:**
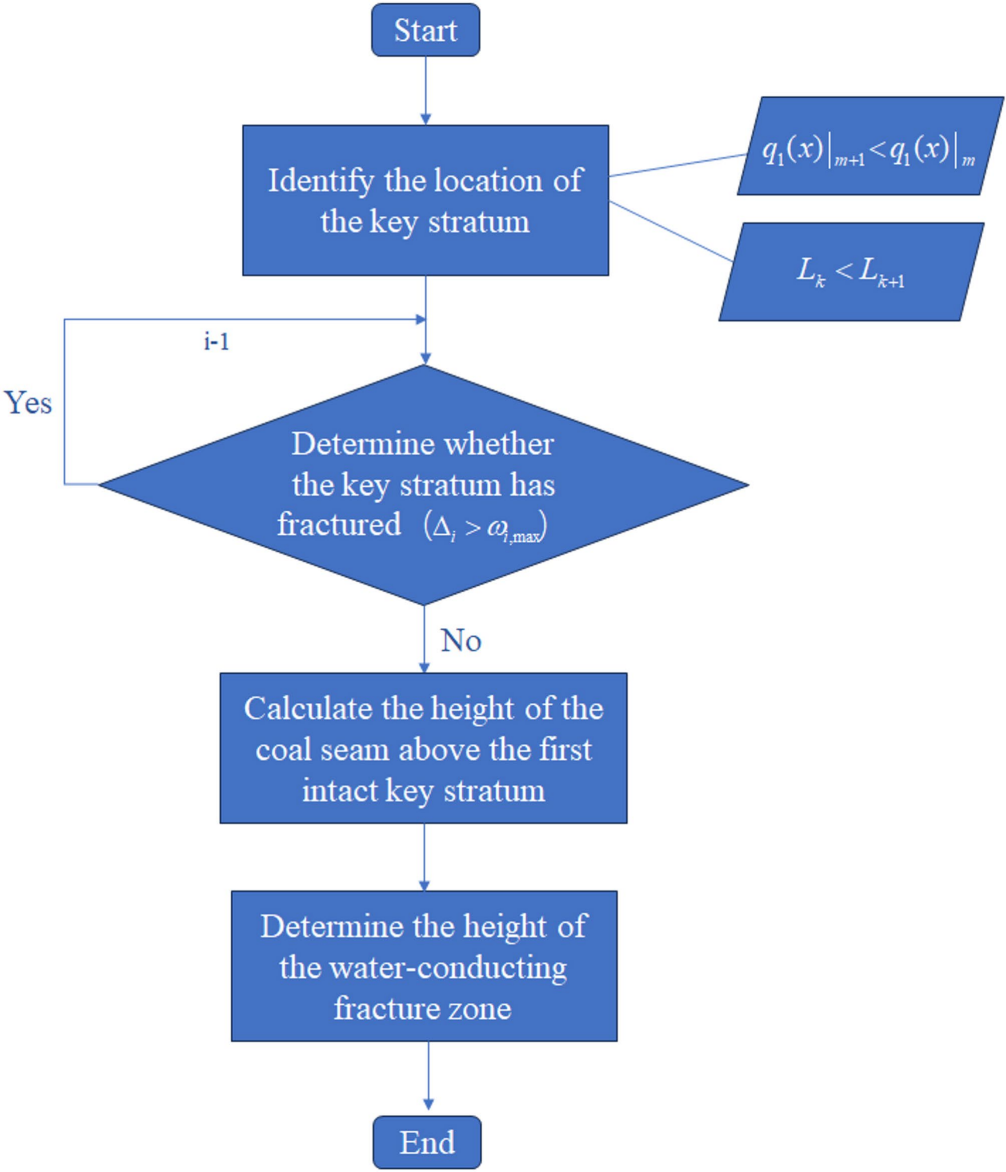
Flowchart for theoretical calculation of water-conducting fracture zone height.

### Analysis of water-conducting zone height caused by thick coal seam mining

The volume of rock strata within the water-conducting fracture zone will increase, and the volume of rock strata above water-conducting zone will not change during the subsidence. The space heights below key strata can be calculated using Eq. (8). Based on the mechanical properties of rock strata obtained from experiments, the calculation results of the space heights below key strata are shown in Table 1. It can be observed that a space exists below five key strata, indicating that all of them will subside. Therefore, whether a stratum will fracture can be determined based on its critical span.

**Table 1:** Bed separation height below key strata.

No.	Rock strata	Thickness (m)	Unit weight (kN/m^3^)	Tensile strength (MPa)	Elastic modulus (GPa)	Key strata	Bulking coefficient	Bed separation height (m)
1	Quaternary	20	18.5	0.01	1		1.05	–
2	Mudstone	35	22.5	0.8	0.58		1.07	–
3	Argillaceous sandstone	48	23.6	0.7	0.7	Main Key stratum	1.07	–
4	Medium sandstone	26	25.1	1.0	1.0		1.10	2.42
5	Mudstone	27	22.5	0.8	1.64		1.07	4.31
6	Medium sandstone	34	25.1	1.0	1.0	Sub Key stratum	1.10	7.71
7	Fine sandstone	20	23.2	1.2	0.68		1.10	9.71
8	Mudstone	15	22.5	0.8	1.78		1.07	10.76
9	Mudstone	15	22.5	0.8	3.36		1.07	11.81
10	Coarse sandstone	20	24.4	0.9	0.9	Sub Key stratum	1.10	13.81
11	Mudstone	25	22.5	0.8	2.56		1.07	15.56
12	Coal seam	15	14.2	0.6	7.58		1.07	1.66
13	Mudstone	38	22.5	0.8	2.56	Sub Key stratum	1.07	4.32
14	Medium sandstone	13	25.1	1.0	1.0		1.10	5.62
15	Mudstone	36	22.5	0.8	5.92	Sub Key stratum	1.07	8.14
16	Coarse sandstone	10	24.4	0.9	4.56		1.10	9.14
17	Mudstone	24	22.5	0.8	5.96		1.07	10.82
18	Coal seam	12	14.2	0.6	4.2		1.07	–

The load acting on key strata is governed by the weight of above coordinated subsidence rock strata, which can be calculated by Eq. (6). There is a space height of 8.14 m below No.15 key stratum, and the critical span of the stratum is 130 m. According to the Eq. (9), the maximum subsidence of the key stratum is approximately 5.13 m, which is less than the available subsidence space height of 8.14 m. Therefore, this subordinate key stratum will fracture, indicating that the water-conducting fracture zone extends above the No. 15 stratum. Additionally, there is a bed separation height of 4.32 m below No. 13 rock stratum. But the maximum subsidence of this key stratum is approximately 4.81 m, which is greater than the available space height of 4.32 m. Consequently, this subordinate key stratum will not fracture, and the strata above it will continue to subside. The complete judgment process flow diagram is shown in Fig. 3. Therefore, the height of the water-conducting zone is approximately 83 m.

### Empirical predication of water-conducting zone height

The empirical formulas for predicting the height of overburden failure are presented in Table 2 when the cumulative mining thickness is less than 15 m. In the table, *H*_*li*_ represents the height of the water-conducting fractured zone, and$\sum M$denotes the cumulative mining thickness of the coal seam. These empirical relationships are derived from extensive field measurements and statistical analyses under conventional mining conditions and are widely used in coal mine water prevention practice. The formulas provide generalized estimations based primarily on mining thickness and roof lithology. The roof strata of coal seams in the study area are mainly composed of relatively weak mudstone interbedded with medium-hard sandstone. From the perspective of safe mining, the roof hardness is conservatively classified as medium-hard. Accordingly, the predicted height of the water-conducting fractured zone is approximately 79.3 m. It should be noted that empirical formulas tend to provide relatively conservative estimations due to geological variability. Therefore, comparison with theoretical analysis, numerical simulation and field measurement is necessary to evaluate their applicability under repeated thick coal seam mining conditions.

**Table 2:** Empirical prediction of the height of water-conducting zone.

Lithology	Formula (m)	Formula (m)
Hard	${H_{li}}=\frac{{100\sum M }}{{1.2\sum M +2.0}} \pm 8.9$	${H_{li}}=30\sqrt {\sum M } {\mathrm{+}}10$
Medium-hard	${H_{li}}=\frac{{100\sum M }}{{1.6\sum M +3.6}} \pm 5.6$	${H_{li}}=20\sqrt {\sum M } {\mathrm{+}}10$
Weak	${H_{li}}=\frac{{100\sum M }}{{3.1\sum M +5.0}} \pm 4.0$	${H_{li}}=10\sqrt {\sum M } {\mathrm{+}}5$

## Numerical simulation of the height of water-conducting zone due to repeated mining

### Numerical modeling

**Fig. 4:**
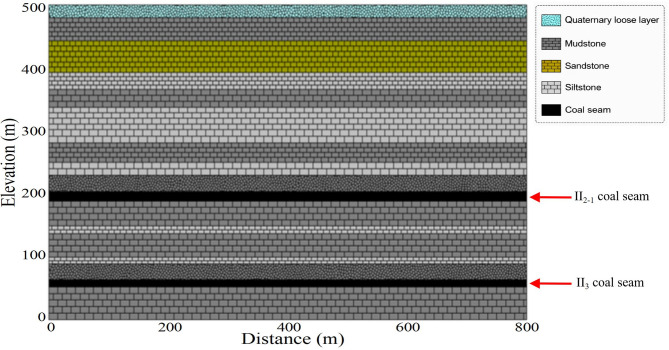
Numerical model of Lingdong coal mine.

Based on the comprehensive stratigraphic column of Lingdong Coal Mine and field-measured stratum thickness data from boreholes in the return airway of 212,302, a numerical model of the complete strata of Lingdong Coal Mine was established by using UDEC software. The model dimensions are 800 m × 500 m and six aquifers and seven aquicludes. The proportion of mudstone in the bedrock column of the model is approximately 60%, consistent with the borehole data from the mine. The left, right, and bottom boundaries of the model are fixed, and a horizontal in-situ stress with a lateral pressure coefficient of 1.0 is applied to the left and right boundaries. The Quaternary loose layer is discretized into voronoi polygons. Similarly, the immediate roof of the coal seam is divided into Voronoi polygons to realistically simulate roof collapse, while other strata are represented as rectangular blocks with horizontal bedding and vertical joints to simulate stratum separation and fracturing. The UDEC numerical model grid division is shown in Fig. 4. In the numerical model, the rock-mass behavior was represented by a block–interface framework. The Quaternary loose layer and the immediate roof were discretized by Voronoi polygons to better reproduce the irregular deformation and caving behavior of weakly cemented strata, whereas the other rock layers were simplified as rectangular blocks containing horizontal bedding planes and vertical joints. This treatment allows the model to capture bed separation, fracture propagation, block interaction and local recompaction during mining-induced deformation.

The physical-mechanical parameters for the numerical model strata were determined through a two-phase approach. For strata between Coal Seams II_2_ and II_3_, core specimens obtained from in-situ drilling underwent laboratory rock mechanics testing, yielding complete stress-strain curves. These curves were used to calibrate the corresponding numerical strata and assign their mechanical properties. For strata above Coal Seam II_2_, parameters were assigned based on compiled geological and geotechnical databases. After parameter assignment, the model was brought to equilibrium under initial conditions, with velocities and displacements reset to zero upon achieving mechanical stability.

Using this calibrated and stabilized model, the mining scheme was designed according to the actual conditions of Lingdong Coal Mine to simulate the evolution of overburden fractures during the extraction process of Coal Seams II_2_ and II_3_ in both the mining face direction and the advancing direction. For the mining scheme along the face direction, the II_2-1_ coal seam was mined first, leaving a 20 m-wide coal pillar. This was followed by mining the II_3_ coal seam in the 212,302 Upper face. The coal pillar left in the II_2-1_ seam is located directly above the central section of the II_3_ 212,302 Upper face. In the advancing direction, the II_2-1_ seam was mined in one continuous operation. The II_3_ seam was then mined incrementally in 20 m advances, totaling 600 m. This approach left the II_2-1_ coal pillar intact while the underlying II_3_ seam was extracted stepwise. The average thickness of the II_2-1_ coal seam is approximately 15 m, and that of the II_3_ seam is 24 m. Sublevel caving was used for the II_3_ seam extraction: the full thickness of the fully mechanized top-coal caving in the upper sublayer was about 12 m, with a cutting height of 3.8 m and a caving height of 8.2 m, resulting in a cutting-to-caving ratio of approximately 1:2. The parameter values listed in Table 3 do not represent conventional hard-roof strata, but were selected to reproduce the weakly cemented characteristics of the mudstone-rich overburden in Lingdong Coal Mine, including low stiffness, low tensile resistance and relatively strong deformation adaptability.

**Table 3:** Mechanical parameters of strata in Lingdong coal mine.

Rock strata	Density (kg m^− 3^)	Elastic modulus (GPa)	Poisson’s ratio	Internal friction angle (°)	Tensile strength (MPa)	Cohesion (MPa)
Surface soil	1850	0.01	0.27	28	0.1	0.2
Argillaceous sandstone	2360	1.4	0.25	35	1.7	11.3
Coarse sandstone	2440	1.8	0.26	30	3.0	4.8
Medium sandstone	2510	2.1	0.25	32	5.0	9.0
Fine sandstone	2320	2.0	0.24	35	4.2	8.2
Mudstone	2250	1.2	0.28	39	0.8	6.4
Coal seam	1420	0.8	0.26	30	0.6	7.1

### Numerical simulation results of overburden failure caused by repeated mining

**Fig. 5:**
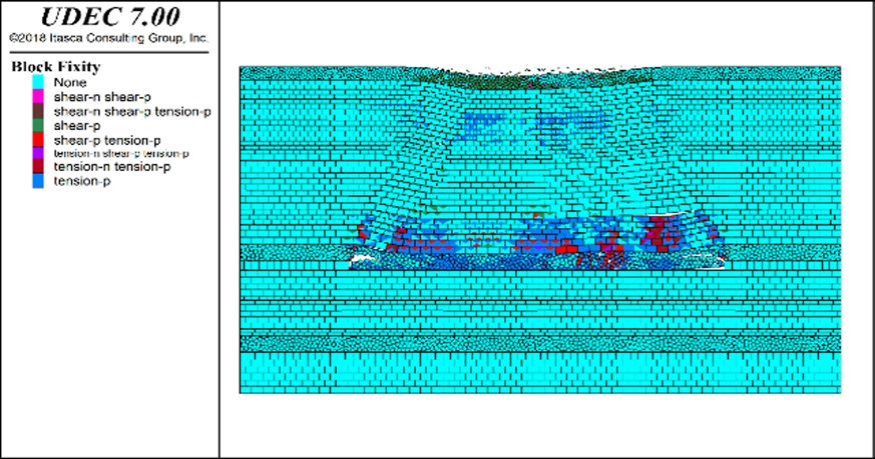
Overburden fracture distribution after II_2-1_ coal seam mining.

**Fig. 6:**
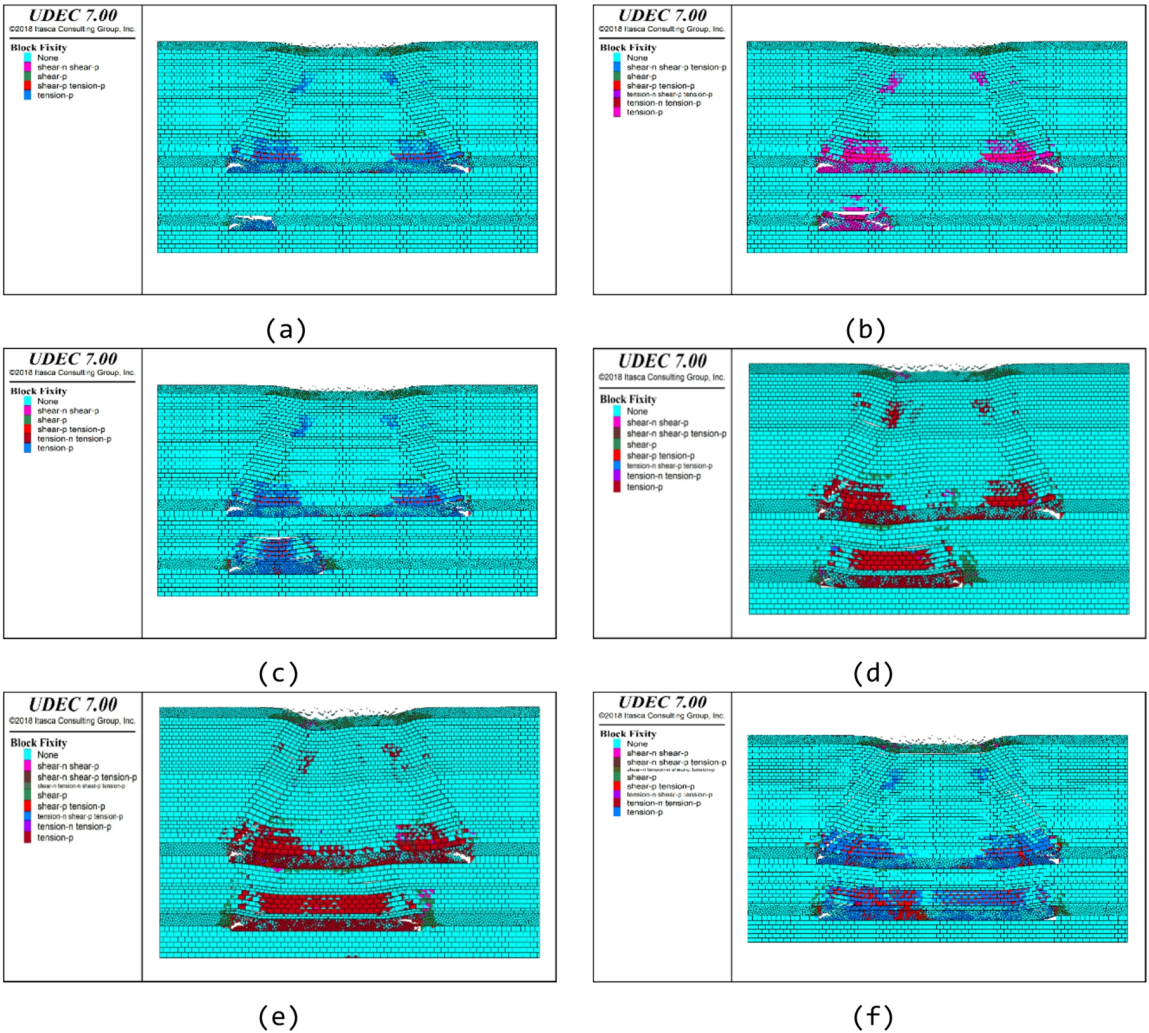
Overburden fracture evolution as II_3_ coal seam advances. (**a**) 100 m, (**b**) 150 m, (**c**) 200 m, (**d**) 300 m, (**e**) 400 m, (**f**) 500 m.

The mining face of the II_2-1_ coal seam was advanced in increments of 50 m. The evolution of fractures at different advance distances is shown in Fig. 5. In the figure, the height of the water-conducting zone in the overlying strata gradually increases with the advance of the mining face. When the mining face advances to 250 m, the height of the water-conducting zone in the overburden essentially reaches its maximum value of approximately 90 m. At this stage, the water-conducting zone connects with parts of the aquifer in the roof but does not extend to the surface and connect with the Quaternary aquifer. When the mining face exceeds 350 m, fractures in the central area of the goaf begin to recompact and close. However, fractures near the open-off cut and the working face remain open and hydraulically conductive. As the II_2-1_ coal seam mining face continues to advance, the height of the water-conducting zone stabilizes and stops further upward expansion, primarily due to the working face reaching a fully mined-out condition. After mining 500 m of the II_2-1_ coal seam, mining of the upper section of the II_3_ coal seam began. Figure 6 illustrates the evolution of overlying strata fractures during the advancement of the 212,302 Upper mining face. As shown in the figure, when the mining face advances 100 m, the immediate roof undergoes initial fracturing, and the fracture height in the overlying strata reaches approximately 24 m. At 150 m of advancement, periodic fracturing of the immediate roof occurs, further propagating fractures upward. When the mining face reaches 300 m, some fractures in collapsed rock masses in the central goaf begin to close due to recompaction, with the water-conducting zone height stabilizing around 73 m. This closure is mainly caused by the gradual load transfer from the overlying strata to the caved rock mass after sufficient mining, which increases the compressive effect in the central goaf and reduces the aperture of earlier mining-induced fractures. Under the mudstone-dominated weak overburden conditions of Lingdong Coal Mine, this process is further enhanced by the strong deformation accommodation capacity of mudstone, which promotes local crack narrowing and partial re-sealing. As mining progresses, the range and degree of fracture closure in the central goaf gradually increase, while fractures near the open-off cut and stopping line remain most developed. By 400 m of advancement, the mining face essentially achieves full mining status, with mining-induced fractures stabilizing in compaction. At this point, the water-conducting zone height stabilizes at its maximum value of about 78 m.

The numerical simulation results indicate that during mining of the upper section of the II_3_ coal seam, the water-conducting zone height gradually increases, with central fractures re-compacting. Water-conducting fractures are primarily distributed above the goaf edges. Upon reaching full mining, the water-conducting zone stabilizes at approximately 78 m, connecting with the sandstone aquifer but not with the upper goaf. Although the water-conducting zone above the upper goaf continued to develop upward, it did not reach the surface or connect with the Quaternary aquifer.

## Field measurement of water-conducting zone height

### Principle of water injection leakage detection method

**Fig. 7:**
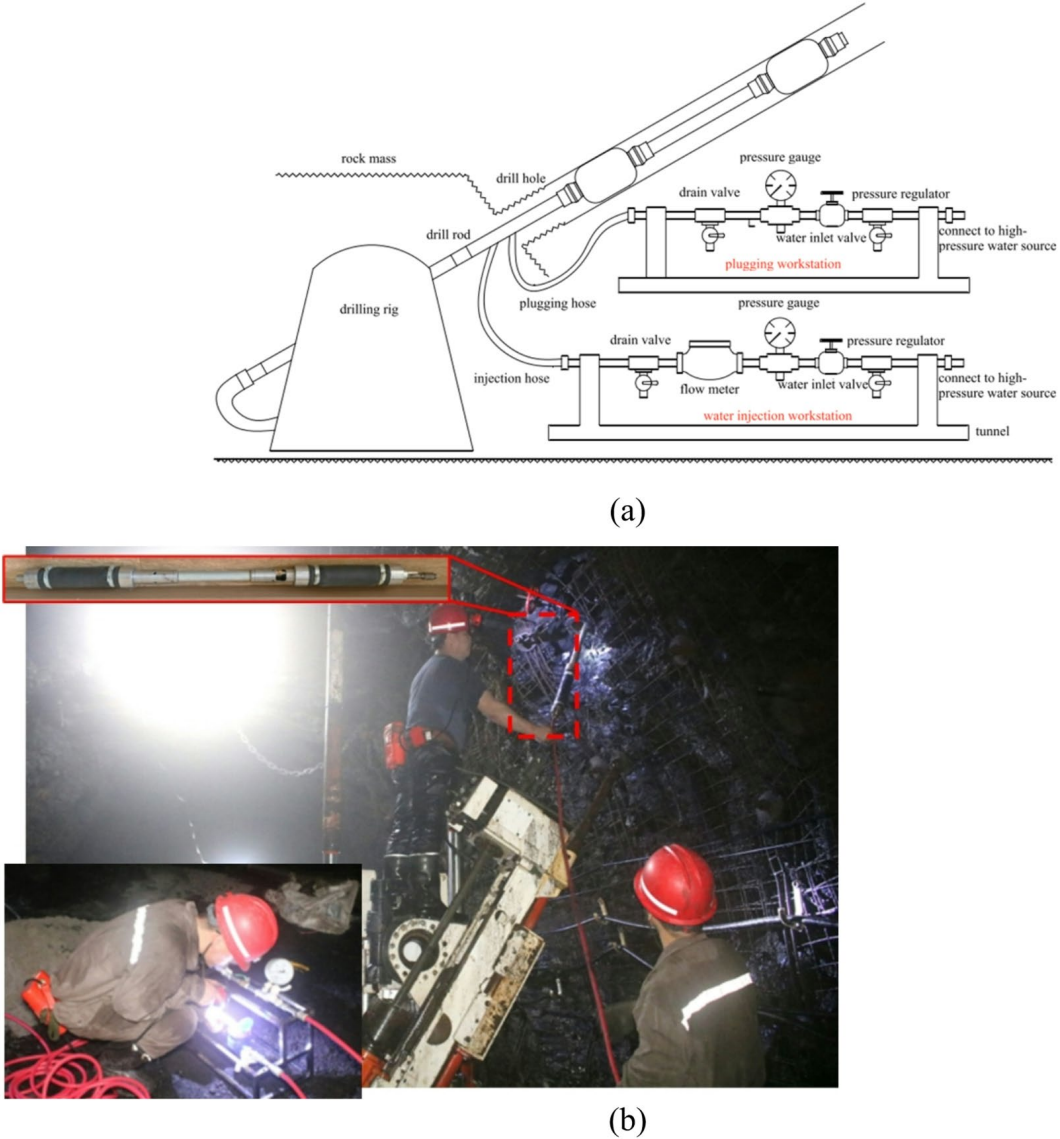
Water injection leakage detection method and field measurement of water-conducting zone.

The fundamental principle of the borehole water injection method involves drilling boreholes at designated angles within the horizontal section or adjacent to the working face of the mining roadway. These boreholes target either the actively mined working face or the previously mined-out area. The borehole depth is designed based on theoretical predictions of the fracture zone height. Upon reaching the target depth, a packer system is installed to perform staged water injection into isolated intervals. By monitoring transient flow rate data during injection for each isolated segment, the fracture characteristics of the surrounding strata can be evaluated. The injection flow rate is highest in the caving zone, followed by the water-conducting fracture zone, and is lowest in the bending subsidence zone. The magnitude of fluid leak-off serves to quantify the development of the fracture zone. The primary equipment for determining the height of the water-conducting fracture zone via the borehole injection method includes an inflatable double-packer system, remote-controlled valves, a real-time monitoring system, integrated with a packer control console and a high-pressure injection pump. Before deployment, the deflated packer assembly is connected to the drill string using the rig and run in hole to the target depth (Fig. 7). Hydraulic pressure is then applied using a triplex plunger pump to inflate the dual packers, creating an isolated test section. Water is injected into the sealed interval through the central conduit, while flow rate and pressure transient data are recorded by the monitoring system. Upon completion of each test cycle, the packers are deflated, and the process is repeated for subsequent intervals. The cumulative fluid leak-off through the borehole wall fractures is quantified using a calibrated electromagnetic flow meter, enabling the correlation between leak-off volumes and the characteristics of the fracture network.

Generally, a low and declining injection rate under pressure indicates competent sealing and limited fracture development in the isolated interval. Conversely, a high and stable flow rate suggests that the injection interval intersects a well-developed caving zone or water-conducting fracture zone. When phenomena such as air entrainment, mud-water outflow, or substantial water volume fluctuations occur, the height of the water-conducting fracture zone can be determined by analyzing the comprehensive flow rate profile.Leak-off tests are conducted at 1-meter intervals along the borehole. The test design and interpretation integrate underground observations, flow rate data, true vertical depth calculations, and insights from inter-hole flow relationships, which are derived from borehole inclination surveys. Three leak-off measurements are recorded per interval to quantify the flow rate. On a roof profile, the borehole trajectory is plotted according to the measured depth and hydrostatically-corrected true vertical depth. Using the borehole trajectory as the vertical axis and injection flow rate as the horizontal axis, a hydrogeological leak-off profile is constructed from the flow rates of each segment. Through comparative analysis of leak-off profiles from multiple boreholes, the height of the overburden water-conducting fracture zone at the target location is determined.

### Designed parameters of drilling holes

**Fig. 8:**
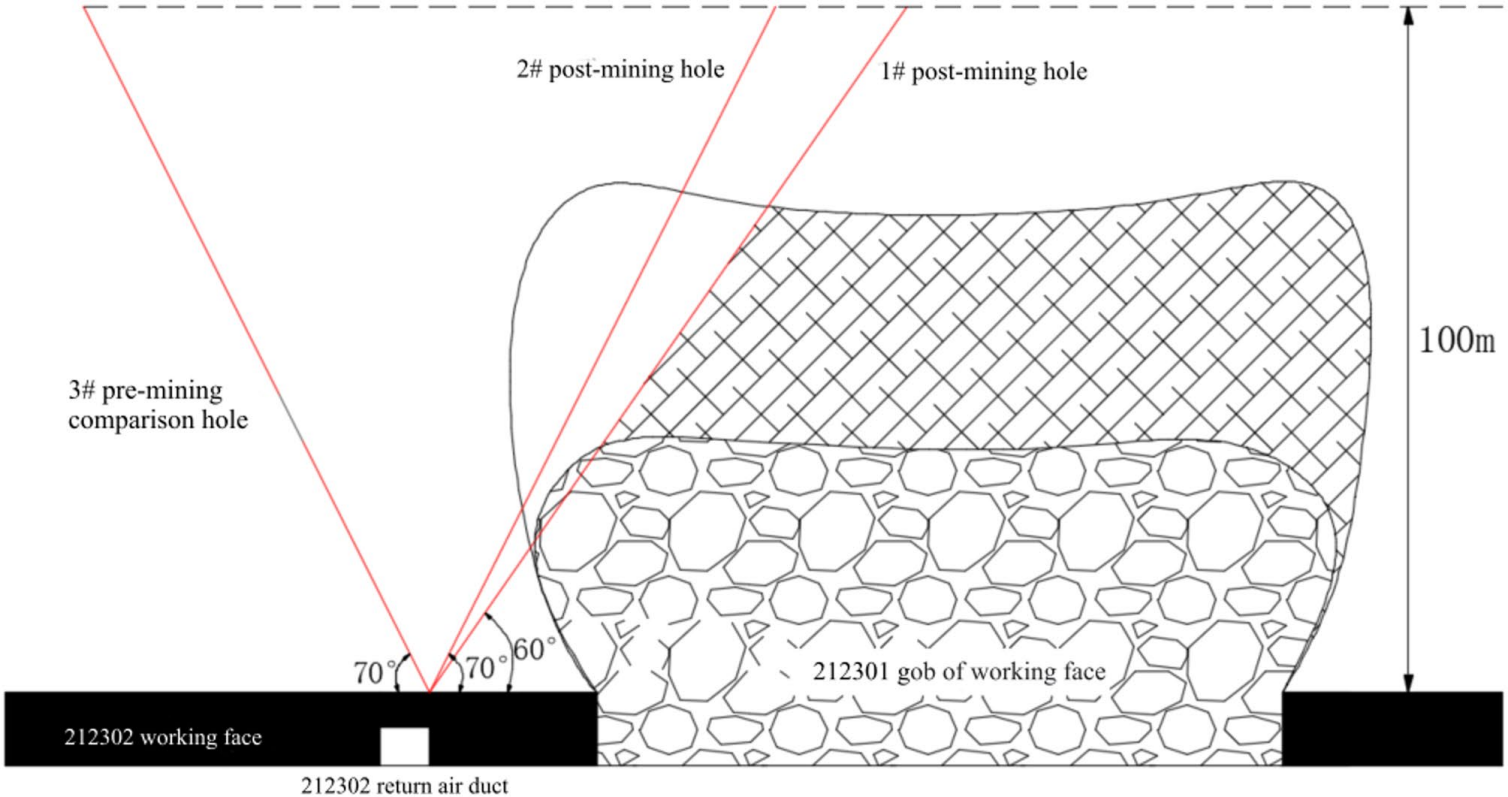
Drilling angle and depth of boreholes.

The overburden fracture zone monitoring array is installed at 1850 m along the No.212,302 return airway, comprising three monitoring boreholes: two boreholes drilled after mining and one baseline borehole drilled before mining. The post-mining boreholes are oriented towards the overlying strata of the No. 212,301 goaf, while the pre-mining borehole is located above the No.212,302 mining face. Borehole parameters are detailed in Table 4. The cross-sectional layout of the “two zones” height observation boreholes is shown in Fig. 8.

**Table 4:** Measured borehole parameters of two bands on the No. 212,302 mining face.

No.	Azimuth (°)	Inclination (°)	Length (m)	Diameter (mm)	Type
1	110	60	115	Φ75	Post-mining borehole
2	110	70	106	Φ75	Post-mining borehole
3	290	70	106	Φ85	Pre-mining borehole

### Measured results of water-conducting zone

**Fig. 9:**
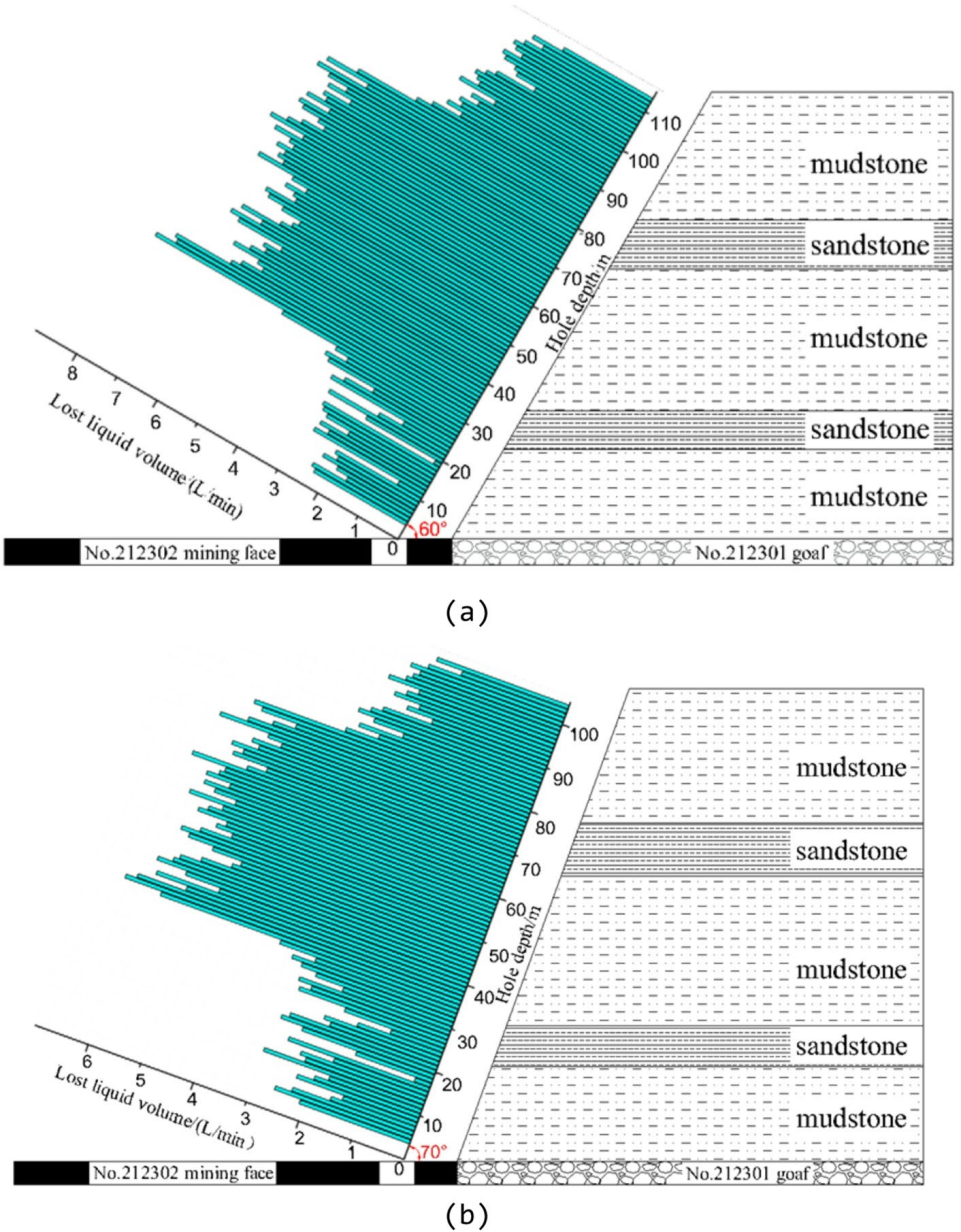
Measured results of water loss at different vertical depths of boreholes. (**a**) No.1 Borehole, (**b**) No.2 Borehole.

**Fig. 10:**
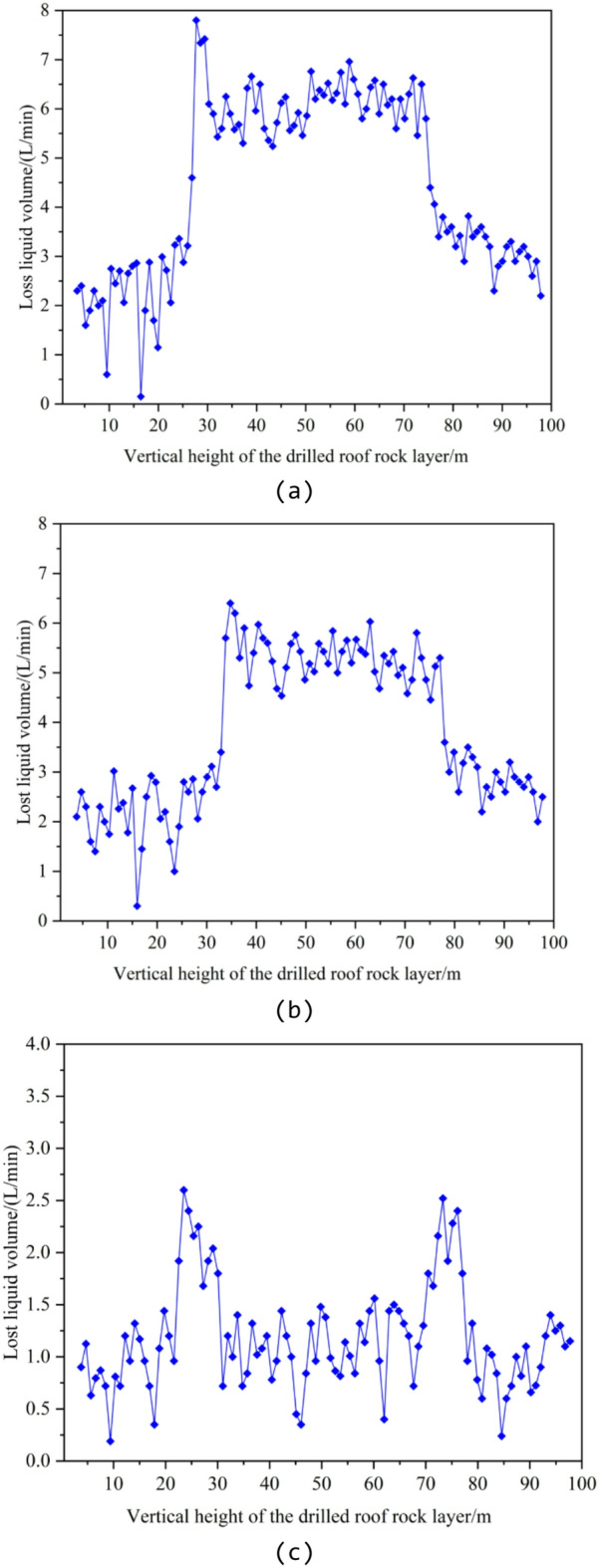
Variation curve of water loss at different vertical depths of the roof. (**a**) No.1 Borehole, (**b**) No.2 Borehole, (**c**) No.3 Borehole.

Borehole #1 (post-mining), Borehole #2 (post-mining), and Borehole #3 (pre-mining) all adopted an upward observation methodology. Measurements in Borehole #1 were conducted to a depth of 113 m, while observations for Boreholes #2 and #3 concluded at 104 m each. The entire observation process strictly adhered to relevant standards, with a stable water pressure maintained above 1.5 MPa. The inflation pressure for the double packers was set to 1.5 MPa. The injection pressure was gradually increased with borehole depth, set to approximately 0.2 MPa above the estimated hydrostatic pressure at each elevation, reaching a maximum of 1.2 MPa. The measured leakage variations at different depths for Boreholes #1, #2, and #3 are presented in the histograms in Fig. 9, which plot the water injection leak-off volume per 1-meter sealed interval against the vertical depth within the roof strata. The leakage rate curves at varying depths are illustrated in Fig. 10.

As shown in the figures, leakage measurements for Boreholes #1, #2, and #3 commenced at a depth of 11 m due to casing installation in the first 10 m of the boreholes, which precluded observations. Specifically for Borehole #1 (post-mining), within the vertical depth range of < 30 m in the roof strata, leakage rates ranged from 0.5 to 3.5 L/min. However, at depths > 30 m, the leakage rate increased significantly to approximately 5.6 L/min, indicating entry into the fracture zone formed by overburden failure from the 212,301 Upper working face. At depths > 74.5 m, leakage decreased markedly and stabilized between 3.0 and 4.0 L/min, defining the upper boundary of the fracture zone. Consequently, the measured height of the overburden fracture zone derived from Borehole #1 is 74.5 m.

Borehole #2 (post-mining) also exhibited leakage behavior consistent with fracture zone development. Within the vertical depth range of < 37 m in the roof strata, leakage rates ranged from 0.5 to 3.5 L/min. However, at depths > 37 m, leakage increased significantly, indicating entry into the fracture zone formed by overburden failure from the 212,301 Upper working face. Beyond 77.1 m, leakage sharply decreased to approximately 3.5 L/min and stabilized thereafter, marking the upper boundary of the fracture zone. Consequently, the measured height of the overburden fracture zone derived from Borehole #2 is 77.1 m.

Borehole #3, as a pre-mining comparison borehole, exhibited significantly lower liquid leakage compared to Boreholes #1 and #2. This is primarily because Borehole #3 was minimally affected by mining activities, preserving the integrity of the overlying strata. However, some leakage (0 to 1.8 L/min) persisted due to inherent fractures within the rock layers. Notably, higher leakage rates were observed in two intervals: from 22 to 30 m and from 70 to 80 m in vertical depth. Field core sampling revealed that these intervals correspond to sandstone layers with higher porosity, while other intervals consist of mudstone with lower porosity and abundant clay minerals, which exhibit self-healing properties, resulting in reduced leakage.

**Fig. 11:**
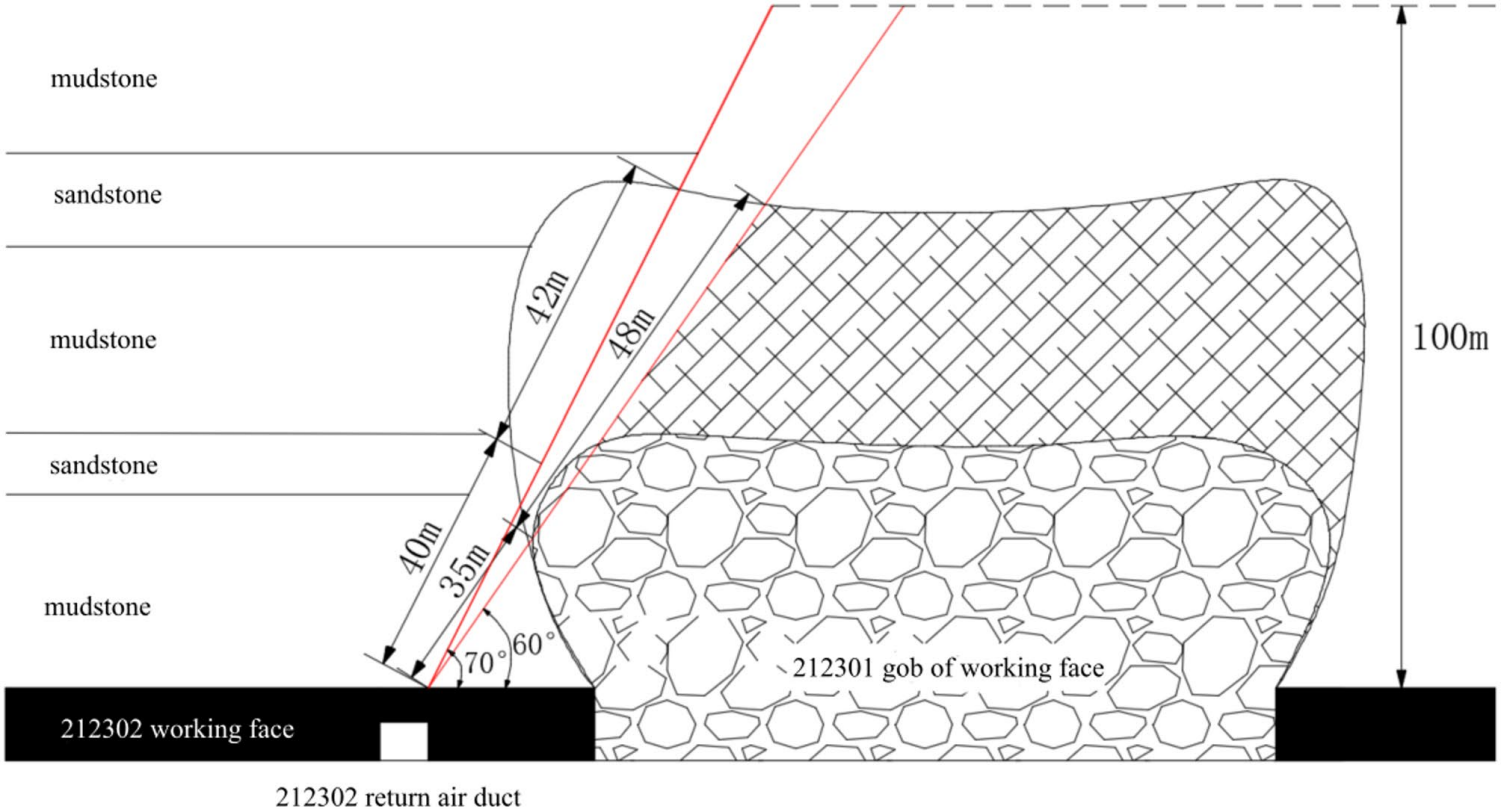
Distribution of water-conducting zone according to field measured results.

The variation patterns of leakage rates with depth in Boreholes #1 and #2 allow the distribution of the water-conducting fracture zone above the 212,301 Upper goaf to be mapped, as shown in Fig. 11. The fracture zone exhibits a saddle-shaped morphology. Borehole #1, with a smaller inclination angle, intersects the upper boundary of the fracture zone closer to the recompaction zone near the goaf center. In contrast, Borehole #2 intersects the upper boundary near the peak region of the saddle-shaped fracture zone. Consequently, the fracture zone height measured by Borehole #2 (77.1 m) is slightly higher than that of Borehole #1 (74.5 m).

The field-measured results of the water-conducting fracture zone height are presented in Table 5. The average measured height of the water-conducting fracture zone is 75.8 m, and the height of the caving zone is approximately 29.4 m. However, the measured height from Borehole #1 is slightly less than that from Borehole #2. The primary reason is that the dip angle of Borehole #1 is smaller than that of Borehole #2, and the fracture zone intersected by Borehole #1 may have undergone more recompaction. In summary, integrating theoretical analysis, empirical predictions, and numerical simulation, the height of the water-conducting fracture zone induced by mining the upper section of the II_3_ coal seam in Lingdong Coal Mine is determined to be between 74.5 m and 83 m. The mining-induced fracture zone from the II_3_ coal seam does not connect to the goaf of the II_2-1_ coal seam. Therefore, water from the upper goaf is unlikely to flow into the II_3_ working face.

**Table 5:** Measured results of caving zone height and fracture zone height.

Number	Caving zone height (m)	Fracture zone height (m)
1	29.4	74.5
2	–	77.1
Average	29.4	75.8

## Discussion

The results obtained from theoretical analysis, empirical prediction, numerical simulation and field measurement show good consistency, which enhances the reliability of the evaluation of the water-conducting fractured zone (WCFZ) under repeated mining of thick coal seams in Lingdong Coal Mine. The theoretical analysis based on key-strata identification and bending deformation gives a WCFZ height of 83 m, while the empirical formula provides a relatively conservative estimate of 79.3 m. Numerical simulation indicates that the WCFZ stabilizes at approximately 78 m after sufficient mining, and field measurements show that the actual fracture-zone height ranges from 74.5 m to 77.1 m, with an average value of 75.8 m. The close agreement among these results suggests that the upward development of mining-induced fractures in the study area is effectively constrained by the weakly cemented mudstone-dominated overburden, rather than following the failure pattern commonly observed in hard sandstone roofs.

This behavior is closely related to the lithological characteristics of the overburden. According to borehole statistics, the bedrock column above the II_3_ coal seam is dominated by mudstone, with an average proportion of about 70.00%. Even in relatively unfavorable local zones, the mudstone proportion remains 41.34%, whereas the sandstone proportion may increase to 58.66%. Such a lithological structure indicates that the overburden is still controlled overall by weak mudstone-rich strata. Compared with hard and brittle sandstone roofs, these mudstone-rich strata are more prone to bending, plastic accommodation and gradual deformation, which consume part of the mining-induced deformation energy and thereby inhibit the excessive upward propagation of fractures. Therefore, the relatively low measured WCFZ height should not be regarded as an underestimation, but rather as a realistic reflection of the deformation mechanism of weakly cemented overburden.

The numerical simulation further reveals that fracture evolution under repeated mining exhibits clear spatial differentiation. During mining of the II_3_ coal seam, the fracture height gradually increases at the early stage, whereas fractures in the central goaf progressively close after the panel approaches full extraction. In contrast, fractures near the open-off cut, the stopping line and the goaf edges remain relatively more developed. This indicates that fracture closure is not merely a geometric compression phenomenon, but the result of the combined effect of overburden load redistribution and recompaction of caved rock masses in the central goaf. As the working face advances and the goaf tends to become fully mined, the broken rock mass in the central area is progressively compacted under the weight of the overlying strata, which reduces the aperture and connectivity of fractures generated during the earlier stage of mining. In addition, the mudstone-rich weak overburden in Lingdong Coal Mine contains abundant clay minerals and exhibits pronounced softening, swelling and deformation-accommodation capacity. These characteristics promote local crack narrowing, partial re-sealing and self-healing behavior, thereby further suppressing the persistence of open conductive fractures in the central goaf. This interpretation is also consistent with the field observations that leakage is relatively larger in sandstone intervals, whereas mudstone-dominated intervals exhibit lower leakage because of their lower porosity and better self-healing characteristics.

From the viewpoint of hydrogeological safety, the combined evidence from lithology, fracture development, and hydrochemical and isotopic analyses suggests that large-scale hydraulic connectivity is unlikely under the present mining conditions. The minimum vertical separation between the II_2-1_ and II_3_ coal seams is about 90 m, while the maximum measured WCFZ height is 77.1 m, leaving a residual protective thickness of approximately 12.9 m. Moreover, hydrochemical and isotopic analyses indicate that the water in the II_3_ goaf is dominated by coal-measure aquifer water, accounting for 85.46%–100%, whereas the contribution from Quaternary water or surface water is only 0–14.54%. This means that direct hydraulic communication between the upper surface/Quaternary water system and the II_3_ working face is weak under current conditions. Therefore, although repeated mining intensifies overburden disturbance and allows the WCFZ to connect with the local sandstone aquifer above the II_3_ seam, it does not cause large-scale connection between the fracture zone and the upper goaf, nor does it lead to direct hydraulic connection with the Quaternary aquifer or surface water.

Overall, the present study demonstrates that the WCFZ in weak overburden under repeated thick-coal-seam mining is jointly controlled by key-strata breakage, bending deformation of weak mudstone strata, goaf recompaction and hydrogeological isolation conditions. The consistency among theoretical prediction, empirical estimation, numerical simulation and field measurement confirms that the proposed evaluation framework is applicable to the geological setting of Lingdong Coal Mine. Under the current mining parameters and hydrogeological background, repeated extraction of the upper section of the II_3_ seam can be regarded as hydraulically safe. Nevertheless, continuous field monitoring is still recommended for subsequent panels to verify the long-term stability of the protective strata and the evolution of water hazards.

It should also be noted that the conclusions of this study are based on the specific geological, mining and hydrogeological conditions of the 212,302 upper panel in Lingdong Coal Mine. Although the combined framework of theoretical analysis, numerical simulation and field measurement improves the reliability of the results, the present numerical model does not explicitly account for time-dependent rock softening, hydro-mechanical coupling, or structural disturbances under more complex geological conditions. Moreover, the weak mudstone-rich overburden is inherently heterogeneous, which may introduce uncertainties in the local fracture response and recompaction behavior. Therefore, future studies should further examine the evolution of the WCFZ under stronger water–rock interaction, longer-term mining disturbance and more complex structural settings.

## Conclusion

To address the development characteristics of the WCFZ in weak overlying strata under repeated mining of thick coal seams, a prediction method for the development height of the WCFZ is proposed based on the key stratum theory. Numerical simulation and field measurement methods were used to study the movement of coal seam overburden and the characteristics of fracture development and distribution, which systematically clarifies the evolution law of the WCFZ in weak overlying strata under repeated mining of thick coal seams. The main conclusions are as follows:

1. The key stratum theory and the critical breaking span of the rock beam are utilized to determine the position of the key stratum within the strata. According to the judgment criteria, when ω_i, max_<∆_i_ are met, the key stratum breaks, and the WCFZ continues to develop upwards. Whenω_i, max_>∆_i_ are met, the key stratum undergoes only plastic deformation without failure, causing the WCFZ development to cease. Consequently, the development height of the WCFZ in the overlying strata of the No. II3 coal seam in Lingdong Coal Mine is determined to be 83 m.​​.
2. Numerical simulation results indicate that after sufficient mining of the No. II3 coal seam, influenced by compaction effects, water-conducting fractures are primarily distributed above the open-off cut (setup entry) and the stopping line, reaching a maximum height of approximately 78 m. The fractures remained disconnected from the overlying goaf. Although mining of the No. II3 coal seam caused the WCFZ from the upper goaf to develop upwards, it did not reach the surface or connect with the Quaternary aquifer.
3. Field inspection results show the development height of the WCFZ is between 74.5 m ~ 77.1 m, with an average height of 75.8 m. This is consistent with the research on the evolution law of overburden fractures. It can be confirmed that the mining-induced fracture zone from the No. II3 coal seam did not connect with the goaf of the No. II2-1 coal seam. This prevents water from the No. II2-1 coal seam goaf from rushing into the working face of the No. II3 coal seam, ensuring no water inrush occurs during mining.
4. The good agreement among theoretical prediction, numerical simulation and field measurement demonstrates the reliability of the proposed evaluation method for determining WCFZ height under repeated thick seam mining conditions. The multi-method verification framework established in this study enhances the accuracy of water-conducting fractured zone assessment and provides a practical reference for water hazard prevention in weakly cemented overburden.

## Data Availability

All data generated or analyzed in this study are included in the published article.
